# Harnessing chemistry for plant-like machines: from soft robotics to energy harvesting in the phytosphere

**DOI:** 10.1039/d4cc06661h

**Published:** 2025-04-03

**Authors:** Isabella Fiorello, Yuanquan Liu, Behnam Kamare, Fabian Meder

**Affiliations:** a Cluster of Excellence livMatS@FIT – Freiburg Center for Interactive Materials and Bioinspired Technologies, University of Freiburg Georges-Köhler-Allee 105 D-79110 Freiburg Germany isabella.fiorello@livmats.uni-freiburg.de; b Surface Phenomena and Integrated Systems, The BioRobotics Institute, Scuola Superiore Sant’Anna Via C. Maffi 27 56126 Pisa Italy fabian.meder@santannapisa.it

## Abstract

Nature, especially plants, can inspire scientists and engineers in the development of bioinspired machines able to adapt and interact with complex unstructured environments. Advances in manufacturing techniques, such as 3D printing, have expanded the range of materials and structures that can be fabricated, enabling better adaptation to specific applications and closer mimicking of natural systems. Furthermore, biohybrid systems—integrating plant-based or living materials—are getting attention for their ability to introduce functionalities not possible with purely synthetic materials. This joint feature article reviews and highlights recent works of two groups in microfabrication and plant-inspired robotics as well as plant-hybrid systems for energy conversion with applications in soft robotics to environmental sensing, reforestation, and autonomous drug-delivery in plant tissue.

## Abstract 2

Nature, especially plants, can inspire scientists and engineers in the development of bioinspired machines able to adapt and interact with complex unstructured environments.

## Graphical abstract

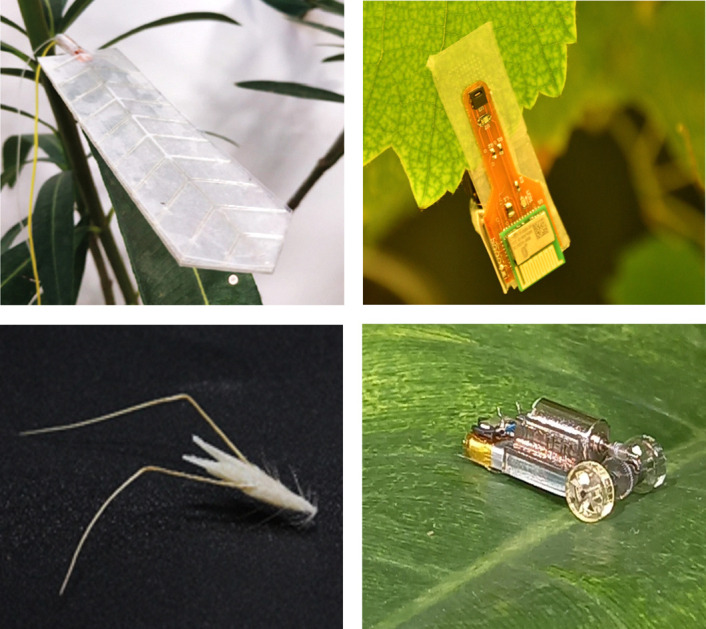

## 1. Materials that drive plant-hybrid and plant-inspired machines

Nature serves as a source of inspiration for scientists and engineers, but it is often also the matrix with which technology interacts with, especially when devices like robots and sensor networks should fulfil tasks in nature. Whenever technology should interact with natural ecosystems, nature- and bioinspired solutions to develop the materials and functions of the systems are advantageous. Organisms often survive by utilizing specialized materials, an important strategy especially for plants, as they must rely on whatever resources are immediately available in their environment. Despite, and indeed because of, the vast disparity between what plants can create from fundamental precursors such as CO_2_, ions, and water, and what engineers and scientists can currently achieve even with the most advanced synthetic methods, we can still draw inspiration from nature's sustainable strategies for actuation, sensing, and energy harvesting^1^—all crucial elements in developing more sustainable robots. To create functionality in artificial technology by mimicking nature^2^ requires thus to include functional materials. Yet, the materials choice that scientists have is often a function of available precursors together with adequate manufacturing techniques that allow to fabricate and mimic the desired structures in complex technologies like robots and machines. Recently, the materials that can be fabricated for example by 3D printing technologies increased significantly.^3,4^ This gives opportunities to better adapt components to the application but also to better mimic natural structures. On the other hand, biohybrid systems combining directly bioderived materials or even living organisms in the devices have increased interest as this can add functionality that would not be possible to easily generate in solely through artificial matter.^5^

Recently, various reviews have explored plants as inspiration for developing soft machines and robots,^6,7^ focusing on the morphology, biomechanics, and adhesive mechanisms of climbing plants and their biomimetic potential,^8–10^ the dispersal mechanisms of plant seeds,^11^ and the bioengineering approaches used to study plants and develop growing robots.^12^

Differently from other articles, this joint feature article highlights the developments of two research groups focussing on approaches based on plant-bioinspiration and plant-hybrid systems ranging from soft robotics, to sensing and energy harvesting directly in the plant tissue. The work will be featured in the context of works from other groups without being a comprehensive review.

Key highlights of this article include:

1. Showcasing the latest developments in bioinspired soft robotics for the phytosphere, with a focus on plant-like machines and related manufacturing processes and materials by Fiorello *et al.*

2. Exploring advancements in energy harvesting within the phytosphere, including spontaneous charging on leaves and materials for harnessing wind and rain energy, along with associated challenges by Meder *et al.*

3. Examining the progress and challenges of biohybrid approaches and discussing prospects for these innovative small-scale machines.

## 2. Bioinspired soft robots for the phytosphere

Over the past five decades, ecosystem health has declined at an alarming rate, with nearly 70% of the planet's biodiversity lost.^13^ The phytosphere plays a crucial role in ecosystem health, by supporting nutrient cycling, soil structure, and biodiversity.^14^ Bioinspired soft robots in the phytosphere could help to monitor these dynamics, promoting sustainable agriculture and environmental conservation.^15^ These robots need to be adapted to unpredictable and complex real-world unstructured environments, fostering nature conservation.^12,16^

In the last decades, various soft robots have been developed by mimicking the biological features of animals and plants^17,18^ including artificial structures and materials and directly organism-derived materials (dead or living) in biohybrid systems.

Examples of animal models in robotics include the caterpillar,^19,20^ octopus,^21,22^ crabs,^23^ worms,^24,25^ snakes,^26^ geckos,^27^ fish,^28–30^ and insects.^31,32^ However, in the recent years, plants have emerged as a crucial model especially for designing self-adaptive and/or miniaturized robots.^8,12^ The most studied plant models in robotics include climbing plants,^33–36^ carnivorous plants,^37^ and a series of self-drilling seeds^38,39^ and/or fruits,^40^ and flying^41–43^ seeds.^11,38,39,44,45^ One promising direction in advancing these bioinspired systems is the development of multimodal designs that can significantly enhance robotic versatility and adaptability to complex environments.^46^ There is no general design approach, and the robots have been prototyped using various manufacturing techniques and materials processing at different length scales, with various materials. Over the last years, we have developed microfabrication techniques that enable precise fabrication, and we highlight here our examples of plant-inspired robots and related manufacturing and material process in Table 1. The different prototypes are described in detail below.

**Table 1:** Overview of our plant-inspired and plant hybrid systems, the materials and fabrication techniques

Plant-like machines	Biomimetic design	Materials	Manufacturing	Application	Bio-hybrid?	Ref.
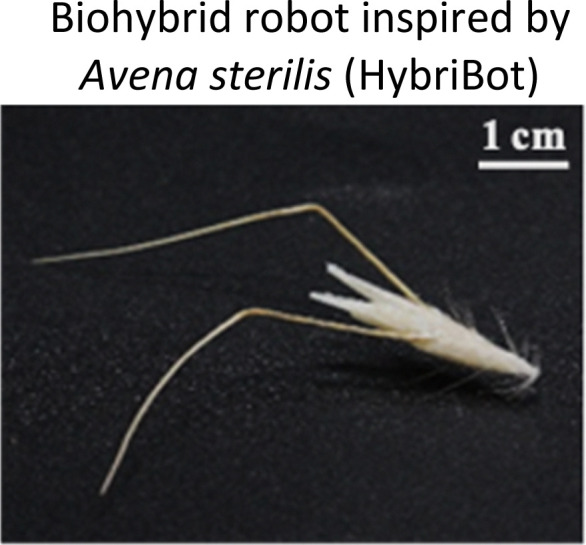	• Two interacting hygroresponsive sister awns as biological motor	• IP-Q photoresist (micromolds)	• Two-photon lithography (nanoscribe photonic GT system)	• Reforestation	Yes	44
	• Functionalized biomimetic artificial capsule	• Flour, biochar		• Precision agriculture		
		• Natural fruit hairs				
		• Natural awns				
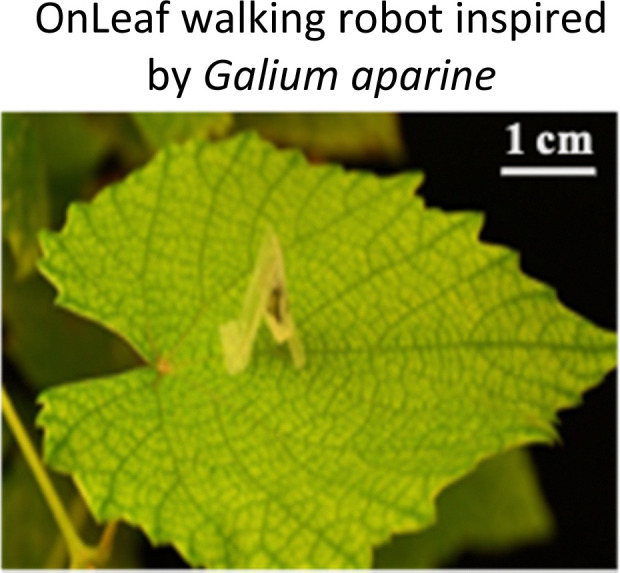	• Direction-based biomimetic microhooks	• IP-S photoresist	• Two-photon lithography (nanoscribe photonic GT system)	• Precision agriculture	No	33
		• Polyethylene terephthalate (PET)	• Laser cutting	• Walking robots		
		• Light-induced fluidic actuator driven by plasmonic heating of nanoparticles (NPs)				
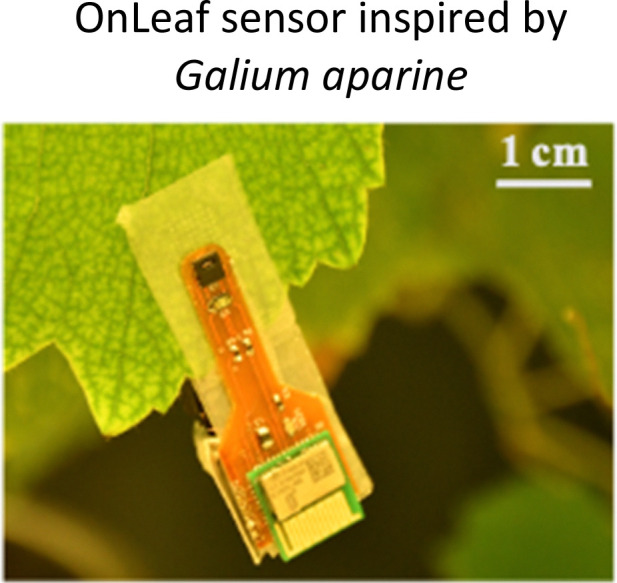	• Direction-based biomimetic microhooks	• IP-S photoresist	• Two-photon lithography (nanoscribe photonic GT system)	• Precision agriculture	• No	33
		• Polyethylene terephthalate (PET)	• Laser cutting	• Precision monitoring		
		• Electronics, battery and sensors (humidity, light, temperature)	• PCB design and assembly			
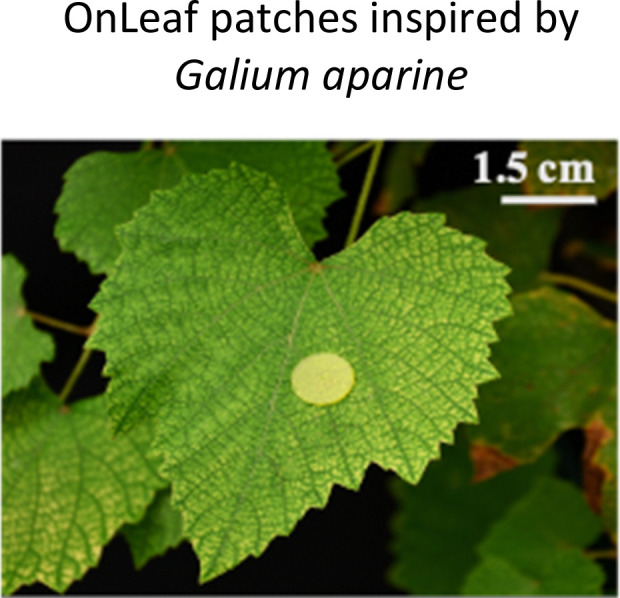	• Direction-based biomimetic microhooks	• Water-soluble isomalt	• Two-photon lithography (nanoscribe photonic GT system)	• Precision agriculture	No	33
			• Micromolding	• Drug delivery		
			• Microcasting			
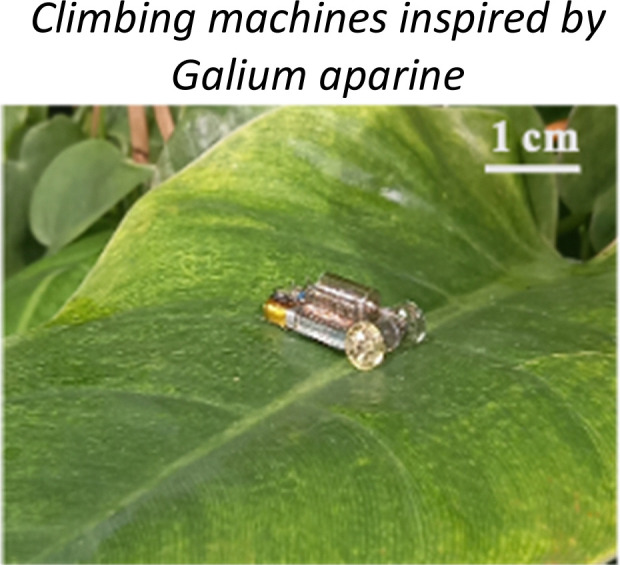	• Direction-based biomimetic microhooks	• IP-Q photoresist	• Two-photon lithography (nanoscribe photonic GT system)	• Precision agriculture	No	47
		• Electronics, motor and battery		• Climbing robots		
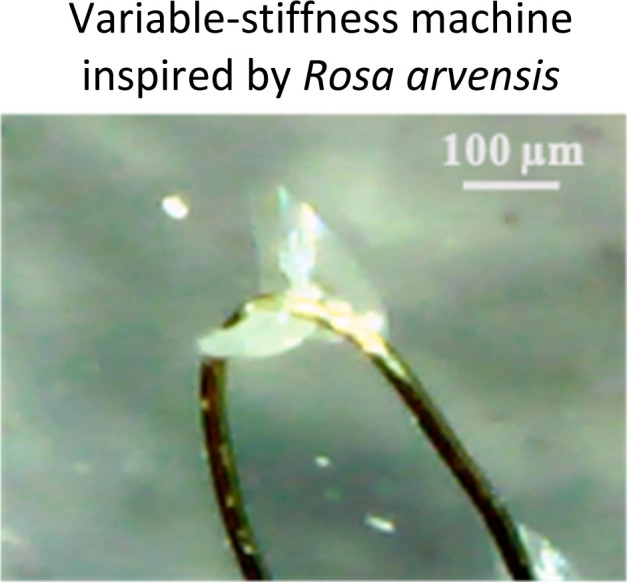	• Direction-based biomimetic microspines	• Polycaprolactone with embedded gold nanoparticles	• Two-photon lithography (nanoscribe photonic GT system)	• Manipulation	No	34
			• Micromolding	• Controllable release		
			• Microcasting			
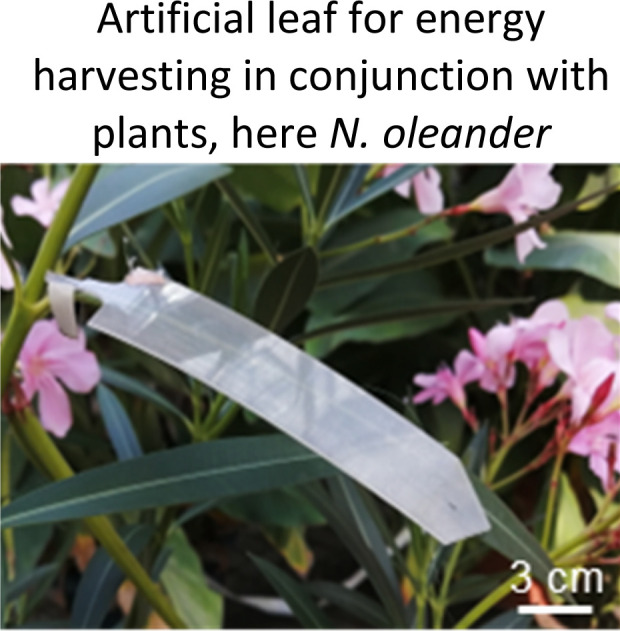	• Petiole and leaf blade	• Multilayer of silicone, indium tin oxide (ITO), polyethylene terephthalate (PET)	• Lamination	• Wind energy harvesting, wind monitoring	Yes	48–50
			• Laser cutting			
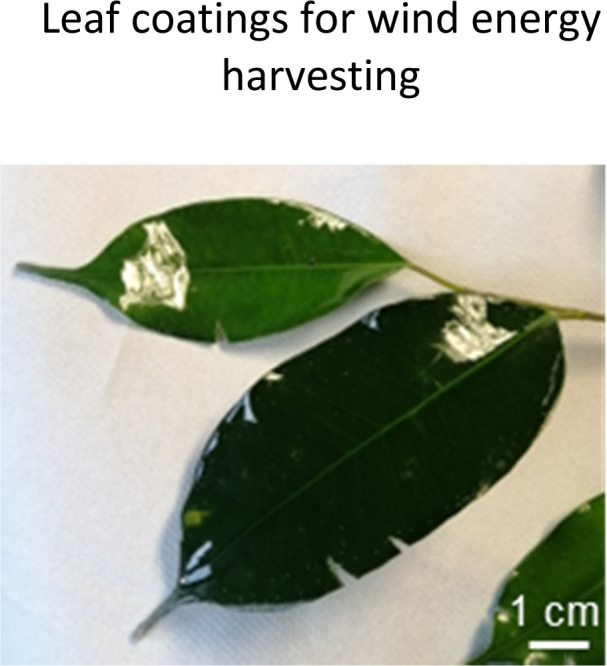	• Coating acts as second cuticle	• Silicone	• Casting, spraying, painting	• Wind energy harvesting	Yes	51
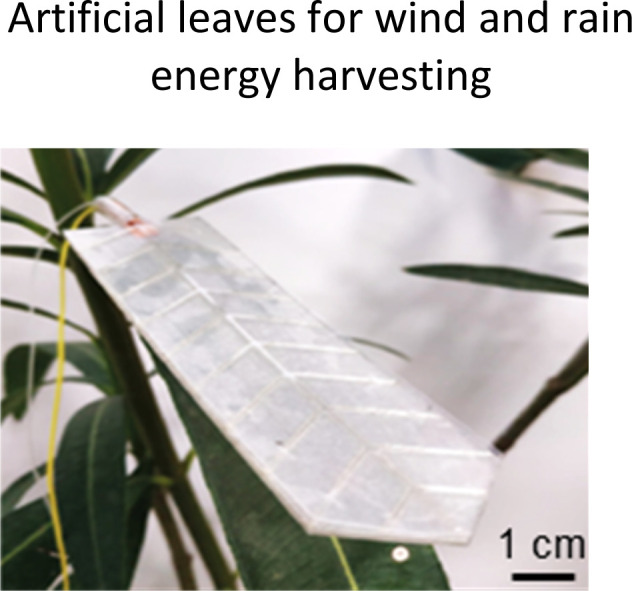	• Petiole and leaf blade	• Multilayer of silicone, ITO, PET, fluorinated ethylene propylene (FEP)	• Lamination	• Rain and wind energy harvesting and monitoring	Yes	52
			• Laser cutting			
			• Mechanical interlocking			
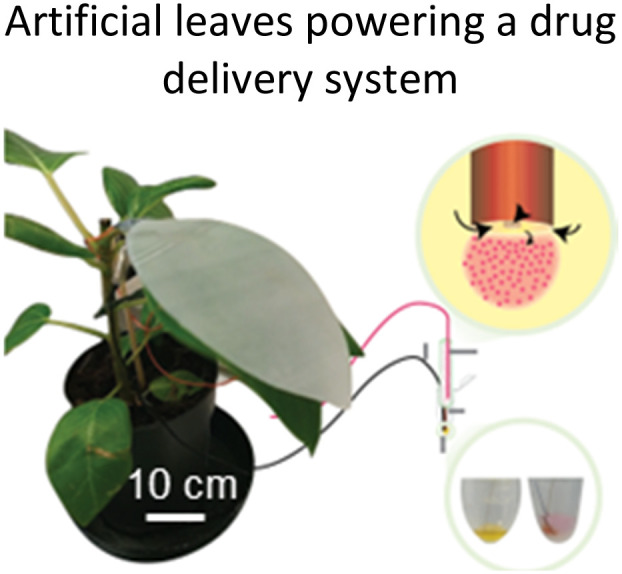	• Petiole and leaf blade	• Multilayer of silicone, indium tin oxide (ITO), polyethylene terephthalate (PET)	• Lamination	• Wind energy harvesting	Yes	53
			• Laser cutting	• Ion delivery		

### 2.1. 3D fabrication of plant-like machines

**Fig. 1:**
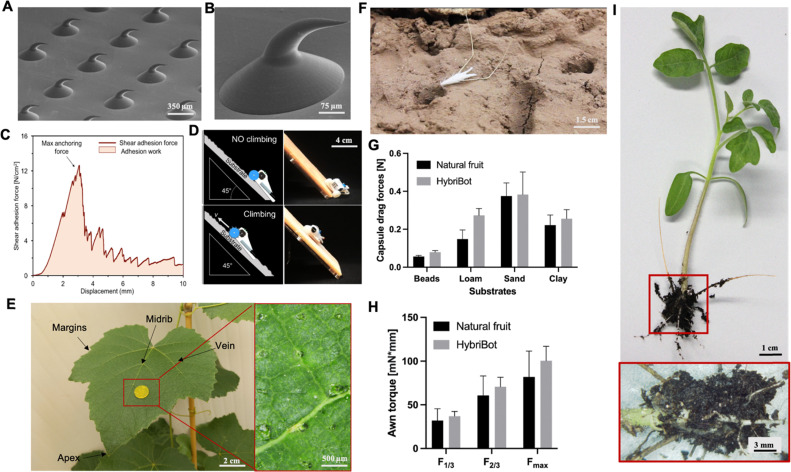
Highlighted works of plant-inspired and/or hybrid machines by the authors. (a)–(e) Micropatterned machines inspired by the ratchet-like attachment mechanisms of the hook-climber *Galium aparine*. These include: (a) and (b) scanning electron microscope (SEM) images of microhooks as arrays (a) and in a single-hook configuration (b); (c) a typical plot of the shear adhesion forces generated by the microhooks on a polyester substrate during measurements in the locking direction; (d) schematic and images of a mobile device without (top) and with (bottom) microhooks climbing on tested rough surfaces, such as artificial skin; [a–d adapted from ref. 35] (e) microhook-based patches for drug delivery into plant vascular tissues, successfully tested for injecting fluorescein molecules into the leaves of *Vitis lambrusca*. Adapted from ref. 33 (f)–(i) Biohybrid machines inspired by the self-dispersing fruits of *Avena sterilis* (HybriBots). (f) A photograph of a HybriBot moving on and within complex soil surfaces; (g), (h) biomechanical characterizations of natural fruits and HybriBots, focusing on capsule drag forces (g) and awn torque (h); (i) successful application of HybriBots for reforestation, demonstrating the germination of tomato seeds from the artificial capsules of the robots. [f–i adapted from ref. 44]. All scale bars are reported in the figure.

Three-dimensional (3D) printing technology plays a key role in the fabrication of miniature bioinspired machines, as it allows to produce complex 3D structures at different scales. In order to mimic plant-structures, often microscale precision is required as functional components in plant tissue range from sub-cell-scale to macroscales. We use additive manufacturing techniques with capability to fabricate miniaturized materials and composites. Among the different powders, inks, or resins based 3D printing techniques,^54–57^ two-photon polymerization (2PP) is particularly powerful and versatile technology,^58^ to fabricate micro/nanotructures from photosensitive materials, including both rigid and soft types.^59^ Recently, Fiorello *et al.* integrated 3D reconstruction through X-ray microcomputed tomography (Micro-CT) to obtain morphological data of the biological structures with micromanufacturing techniques such as two-photon lithography, molding, and casting using biodegradable materials to create biohybrid machines inspired by wild oat fruits (HybriBots).^44^ HybriBot combines actuation of oat fruits using plant-derived materials with engineered 2PP-printed seed capsules that can adapt to environmental changes by navigating soil irregularities and digging into the soil, mimicking the dispersal movements and biomechanical performance of natural fruits. It achieves comparable capsule drag forces of up to ≈0.38 N and awn torque of up to ≈100 mN mm^−1^ (Fig. 1(f)–(i)). Moreover, the authors could functionalize the capsule of HybriBots with fertilizers and/or other seeds, opening potential applications for reforestation and precision agriculture (Fig. 1(f)–(i)).

Another work of the authors introduces the micromanufacturing *via* two-photon lithography of climbing plant-like miniaturized micropatterned machines with microhooks for reversible attachment. Again, inspiration of the leaf microstructures was taken by extracting morphology of microhooks exposed on the organs of *G. aperine*. The resulting micropatterned surface can be used in machines to attach strongly and reversibly over various micro-rough natural and artificial surfaces (including fabrics,^35^ skin tissues,^35^ and leaf tissues^33,47^), by reaching shear locking forces up to 14 N cm^−2^ (Fig. 1(c)). This plant-inspired material systems have been successfully applied to climbing robots, controllable manipulators,^34^ leaf sensors for *in situ* monitoring,^33^ and even leaf patches for drug delivery into leaf vascular tissues^33^ (Fig. 1(e)) as described later in detail. This demonstrates potential for achieving new tools in soft robotics and for precision agriculture, that may fulfil tasks in the preservation of our environments and the phytosphere.

Although 2PP printing enables highest precision, also other techniques like digital light processing (DLP), polyjet, direct ink writing, fused deposition modelling, or moulding techniques can be sued to mimic plant functions in robotic systems. The resulting materials need to be compatible with the environment and interact with it specifically, *e.g.*, interactions with ambient humidity or sunlight for actuation.

Fabrication methods and material choices played a crucial role in replicating dynamic, bioinspired behaviors. Light-curing 3D printing (DLP) used thermosensitive polymers with tunable memory effects—achieved by controlling printing temperature and layer height—to mimic the deformable petals and curling of natural organisms.^60^ Polyjet technology's ability to print both flexible and rigid materials in full color enabled the creation of hydrogel–elastomer composites that transformed from circular to star shapes, replicating cactus-like structures and achieving responsive motion under environmental cues.^61^ Direct Ink Writing (DIW), suitable for soft materials and low-temperature deposition, allowed the production of lightweight, porous cellulose acetate structures resembling Tragopogon fruit,^41^ enabling prolonged airborne suspension and environmental monitoring. Finally, Fused Deposition Modeling (FDM) facilitated the fabrication of pinecone-inspired PEEK robots with gradient crystal structures and surface roughness, leading to reversible, solvent-responsive deformations and robust mechanical performance.^56^ In each case, careful material selection and printing parameters were key to reproducing the adaptive, responsive functions found in nature. Yet, new approaches are required to enhance the adaptability of materials, enabling functionalities such as controlled biodegradability, electrical conductivity, and programmability. Current works on biodegradable electronics are contributing to this issue and lack of functional materials, and can be used to prototype sustainable biodegradable fully-integrated robots, or to control the robot lifetime.^62–65^

### 2.2. Chemistry of materials for plant-like machines

The selection of materials is a central factor in the development of plant-like machines. To achieve flexible movement and adaptive properties that mimic nature, these materials often have unique physicochemical properties such as environmental and stimuli responsiveness, programmability, durability, and high elasticity.^41,44,60^ Carefully selecting precursors and integrating them with 3D printing technology is the key to obtain plant-inspired and plant-like behaviours.

Photosensitive resins are essential for 2PP 3D printing, for example, we recently mimicked the ratchet-like attachment mechanism of *Galium aparine* by utilizing 2PP (see Section 2.1). One method involves directly printing micropatterned hook-like rigid structures using IP-S photoresists on a flexible Mylar® sheet as a substrate. The resulting flexible micropatterned materials could be precisely cut (*e.g.*, *via* laser cutting) and subsequently integrated into climbing miniaturized robots, leaf-walking robots, or sensors (Fig. 1(a)–(d)). Another method involves directly printing micropatterned wheels and/or robotic mechanical components with plant-like organs such as microhooks using photoresists like IP-Q on a rigid silicon wafer substrate.

Furthermore, by combining 2PP with PDMS micromolding and casting favorable materials, we demonstrated the feasibility of prototyping soluble, biodegradable, and/or flexible plant-like machines with microhooks or spines. For instance, we cast a composite of polycaprolactone and embedded gold nanorods in a PDMS mold obtained from replicating 2PP-printed plant-like microhooks to create micropatterned materials achieving tuneable stiffness *via* untethered and controllable plasmonic heating-induced phase changes in the polymer. Additionally, we used isomalt with embedded fluorescein molecules to cast 2PP-printed and PDMS replicated molds and fabricate micropatterned hooks that first interlock with biological tissue and subsequently dissolve as a proof-of-concept for *in situ* drug delivery applications into plant vascular tissues.

As anticipated above, we have exploited 2PP also to prototype our HybriBots. Using Micro-CT, we created a detailed 3D reconstruction of the *Avena sterilis L.* capsule, which guided the design of two complementary miniaturized molds fabricated *via* 2PP using the IP-Q photoresist. After casting and coating, we developed biohybrid machines consisting of a biodegradable, edible capsule made from flour-based material, coated with natural hairs of *Avena sterilis L.*, and equipped with two humidity-driven natural sister awns connected to the capsule.

## 3. Soft materials for energy harvesting in the phytosphere

The question of energy autonomy and potential power sources for the machines, sensors, and data communication becomes especially critical when deploying devices and robots in environments far from a power grid like plant-dominated ecosystems.^66–68^ In other words, how could robots harness energy when operating and collecting data in the canopy of a forest or in a grass blade-crowded meadow? How could devices be recharged in such an environment? Several new actuation strategies of small-scale robots have been developed^69–71^ in the last years based on integrated chemical or physical energy sources and energy conversion mechanisms^72–74^ using environmental energy sources such as light,^75–78^ humidity,^79–82^ and temperature changes.^83^ Instead, data collection, sensing, or even computing yet typically requires electricity. Harvesting electrical energy from the direct environment is an approach that could help in satisfying these energy needs. Sustainable solutions could come from plant-derived materials^84^ and those intrinsically available in the environment, for example those externally exposed on living plant surfaces (the phytosphere).^85^

**Fig. 2:**
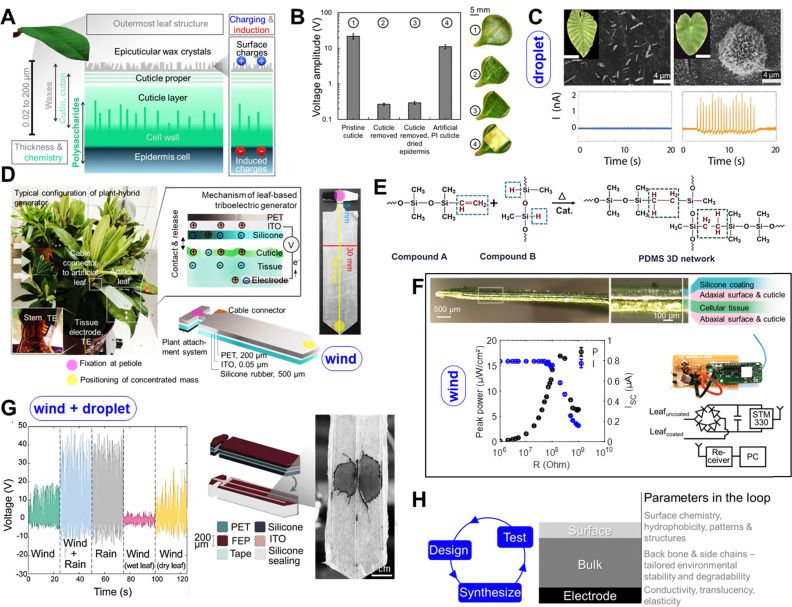
Plant-hybrid approach for energy conversion and harvesting. (a) overview of the structure of the plant cuticle which can be used together with tuned artificial materials as a platform to convert mechanical energy into electrical charges. (b) the effect of the cuticle on the voltage amplitude generated in a *Sedum makinoi* leaf upon contact with silicone rubber: removing the cuticle decreases the generated voltage, adding an artificial polyimide cuticle largely recovers the energy conversion capability showing the crucial contribution of the outermost plant surface on the energy conversion mechanism. Adapted from ref. 85. (c) Current signals measured when water droplets hit the leaf surface. On superhydrophobic leaves of *C. antiquorum* (right) the current peaks are two magnitudes higher than those on *A. macrorrhiza* (left) likely due to the different epicuticular wax layers shown in the SEM images above (scale bars in the insets showing photos of the leaves are 10 cm). Adapted from ref. 86. (d) artificial leaves for plant-hybrid wind energy harvesting exploiting contact electrification on the leaf surface and on the artificial leaves that are installed on the plant leaf and create energy converting contact-and-release motion together with the plant leaf in wind producing sufficient electricity to power LEDs and wireless sensors platforms. Adapted from ref. 50. (e) molecular structure of silicone PDMS, one of the materials that create strong contact electrification with leaf surfaces. Adapted from ref. 89. (f) Microscopy image of silicone-based leaf coatings that can convert the contact between fluttering leaves into electricity, power output of an uncoated leaf in repeated contact with a coated leaf, and circuitry for energy harvesting and powering the depicted wireless sensor. Adapted from ref. 51. (g) Upgraded artificial leaf for plant-hybrid wind and rain energy harvesting. The wind-energy harvesting structure is upgraded on the surface for converting droplet impact into electricity. Voltage peaks under different conditions are shown and an image of a droplet landing on the leaf. Adapted from ref. 52. (h) Material parameters that can be tailored through their chemistry to improve the artificial leaf and tune its energy conversion efficiency and power output. The blue pill-shaped boxes in (c), (d), (f), and (g) indicate which mechanical energy source is converted into electricity by the plant-hybrid systems.

Our research in the last years showed that the structures and materials on the outer plant surface and especially in the epicuticular region, could excellently be exploited for mechanical-to-electrical energy conversion through solid–solid and liquid–solid contact electrification.^49,51,52,85,86^ The prototypes are highlighted in Table 1. Featuring our technology requires a deeper look into the intrinsic properties of the outer surface of plants and especially leaves. The surface structure of leaves is unique, comprising not only physiological elements like well-known stomata but also precisely tuned polymers and *per se* chemically fascinating components. Above the epidermis cells, the leaf cuticle establishes the surface layer as depicted in Fig. 2(a). It is a composite consisting of a hierarchical polyester network made of cutan and/or cutin, a C16–C18 hydroxy and epoxy fatty acid, incorporating polysaccharides like pectin or cellulose, and different waxes.^87^ The top, outermost surface is typically a layer of epicuticular waxes, with different long-chain aliphatic and cyclic compounds like hydrocarbons and functional groups like –OH, –COOR, –CHO, carbonyl groups.^88^

The epicuticular waxes are directly interacting with the air environment. The exact composition can vary significantly between plant species and the epicuticular wax layer can be more or less dense and essentially contributes to well-known mechanisms like self-cleaning, super-hydrophobicity and the Lotus effect.^90,91^ Especially the epicuticular waxes and their structure have a significant impact on the development of spontaneous electrical charges on the surface upon contact with solid materials and leaves – through contact or triboelectrification as our results show.^85,86^ While triboelectric charging appears on most materials,^92^ it is surprising that the charge formation is significantly affected by the epicuticular wax composition and especially structure. Indeed, several researchers try to copy leaf's wax structures to enhance artificial energy harvesters like triboelectric nanogenerators.^93–99^ Another important factor is, that the cellular tissue under the purely polymeric cuticle is ion-conductive consisting of water and ions.^85^ It can hence be used as electrode to analyse and harvest the charges (schematized in Fig. 2(a)). We showed that removing the cuticle from leaves strongly reduces the voltage measured in the plant tissue *versus* ground and thus the charging (Fig. 2(b)). Using an artificial polyimide layer as “artificial cuticle” increased charging again but did not reach original levels with the pristine cuticle, although polyimides are known to cause high charging in artificial triboelectric nanogenerators.^85^ Pristine plant leaves have been used in various solid–solid and liquid–solid triboelectric nanogenerators (TENGs) leading to voltage and power outputs of >200 Volts and >5 μA per single leaf^48,49,51,85,100–105^ and modified plants have been used for energy storage.^106,107^

We made interesting observations in epicuticular charging when measuring the charge as function of cuticle composition and wax layer structure when water droplets hit the leaf surface.^86^ We used a model of two plants from the family Araceae that have a similar leaf structure and tissue impedance but with totally different surface wax coating densities. We observed first that both leaves develop surface charges when droplets hit the surface. However, leaves with a less dense wax layer of *A. macrorrhiza* became less charged (Fig. 2(c)). This could be due to two effects, one could simply be that wax crystals are the charging sites, for example their tips, and fewer wax crystals lead to fewer effective sites for contact electrification. Another role, could also play a remaining water layer that could compensate charges, enhance charge dissipation, or hinder intrinsic charge formation. Instead on superhydrophobic leaves of *C. antiquorum*, the current peaks measured in the tissue were 10–100 times higher confirming increased charging.^86^ This could be due to the complex chemical composition and the nanoscale structure. Thus, we performed an experiment in which we maintained the surface chemistry and changed the overall surface structure by a gentle thermal treatment, that melted and flattened the wax layer but did not affect the general composition as confirmed by infrared spectroscopy.^86^

Charging decreased by a factor of 10 when melting the wax structure although it did not reach the lower values of the hydrophilic leaves of *A. macrorrhiza*. Due to melting and flattening the wax layer, also a change in the contact angle was expectedly caused from 150° to 80° which could again lead to a more significant remaining water layer affecting the charging.^86^ While this needs to be further clarified in detail in ongoing work, the experiment showed that both, chemistry and but especially also the wax structure affect charging significantly.

### 3.1. Collecting and harvesting charges in the phytosphere – wind energy harvesting

Confirming that charging on leaves surfaces can be significant, one needs to try to find modes to harvest and collect the energy. Our first inspiration came not from plants but from artificial triboelectric nanogenerators where energy conversion happens due to a reoccurring contact and separation motion.^108–110^ Yet, we wanted to exploit the leaf surface and harvesting in the tissue acting as electrode in order to make use of the passive motion of leaves fluttering in wind as energy source. To address the latter, we developed artificial leaves (Fig. 2(d)) that can be individually installed on the plant leaf to create effective contact and separation motion together with the plant leaf.^48–50^ These artificial leaves need to be coated with a material that enhances contact electrification with the leaf surface and the epicuticular waxes. The well-known material dependency of contact electrification and the triboelectric series helps to select appropriate materials but empirical tests are necessary especially when complex materials like leaves are tested. We thus exposed the leaves to a series of materials following the triboelectric series and found that especially silicones lead to highest charging,^85^ even higher than fluorinated polymers like PTFE often performing best in artificial TENGs, when tested under same impact force. This is on the one hand due to the specific surface structure of silicones as shown in Fig. 2(d). The polar Si–O bonds in the backbone, combined with the carbon components, its dielectric and hydrophobic properties^89^ could stabilize charged states. In addition, also to the sticky fragile nature that could break up matter on the leaf and silicone surface. Moreover, the elastic deformation could help in increasing effective contact area for charging on the microscale compared to more rigid materials.^49^ The charging mechanism could not be clarified yet in detail, also due to the complex composition of leaf surface, and further investigations are necessary. Which materials are best for energy harvesting is still a major question on artificial TENGS, especially when questions like biodegradability and avoidance of problematic fluorinated polymers should be addressed.^111–116^ Despite that, we focussed our investigations on silicone as excellent material with advantages for plant-hybrid technology due to its softness (→ it does not damage the tissue during mechanical contacts tissue) and its transparency (→ to not affect photosynthesis). Moreover, our artificial leaves are lightweight (∼3 g) to avoid damages by the weight of the device.^117^ To further assess the effect of artificial materials like silicones on leaves, we conducted a one-year experiment with a *Ficus* plant, where we coated all its leaves on the top with a silicone elastomer film. Over this period, we monitored transpiration and growth and observed no visible damage.^51^ We exposed hybrid plants consisting of plants and artificial leaves to different wind speeds and directions under well-controlled conditions in a phytochamber with controlled wind speed, humidity, temperature and light and observed the power output varying the parameters, especially wind speed with a focus on low wind speeds (below 5 m s^−1^).^48–50^ Moreover, we tested how to avoid the artificial leaf and directly coat leaves with a thin silicone layer (Fig. 2(f)).^51^ These approaches resulted in significant power generation, that is wind speed-dependent, affected by external conditions like humidity (causing a reversible decay of charge generation), and by the mechanical and aeroelastic behaviour of the artificial components while interacting with the leaves. Importantly, energy harvesting using the phytosphere directly for energy conversion, does not require complex materials, as mentioned, only a silicone layer and a transparent electrode are sufficient. The peak power generated so far reaches up to 17 μW cm^−2^ and is sufficient to directly power LEDs (100 with a single leaf). For energy storage, capacitors are used, or the charges generated are directly used to power LEDs, digital meters, or act as sensor signal. Moreover, we showed recently that only 8 artificial leaves can repeatedly power an environmental humidity and temperature sensor including 868 MHz wireless data transmission autonomously over several days with data transmission intervals of ∼40 min and an RMS power of 6 ± 0.6 μW at a wind speed of 3.3 m s^−1^.^49,51^ Considering that the 8 leaves have a total apparent surface area of ∼0.03 m^2^, the RMS power normalized for the area is ∼200 μW m^−2^. This is an output similar to recent low-wind artificial triboelectric and piezoelectric wind harvesters.^118,119^ A further comparison of the power output of plant-hybrid energy harvesters with other energy harvesters and the energy consumption of electronics can be found in the articles.^49,51^

Yet, improvements in the materials ranging from surface micro-nanostructuring, surface chemistry, to the mechanical behavior as also forecasted by our models^117^ describing the elastic deformation under a wind load, show that further improvements can be achieved addressing this multidisciplinary aspects in addition to selecting the best plant species. Another approach that we did already to improve the output is implementing multisource energy harvesting by adding the capability to convert other energy sources in addition to wind by the plant hybrid systems.

### 3.2. Adding additional energy sources – multisource energy harvesting through materials and plant-hybrid systems

In addition, to the wind energy harvesting described in detail in the previous section, other energy sources can be harvested as well. As seen mentioned before, charges can be generated by droplets and this has been particularly shown on artificial materials based on fluorinated polymers like FEP and PTFE.^120,121^ The currents that we observed in the superhydrophobic leaves after droplet impact were too small to be used for energy harvesting but artificial materials can enhance charge formation when water droplets interact with surfaces. This has been extensively presented with different fluorinated polymers^122^ which enabled significant energy harvesting from surface charging during interactions with single droplets and exploiting surface electrodes that contact the droplets.^120,121^ Based on these results, we could implement a surface structure on our artificial leaf to establish and equip it with the function of rain drop energy harvesting in addition to wind energy harvesting.^52^ It was required to add a FEP layer on top of the PET layer and ITO surface electrodes on top of the FEP layer as depicted in Fig. 2(g). Together with the central electrode also used during wind energy harvesting, the droplet spreading on the FEP surface creates charges but also connects to the surface electrode changing its area and the device was capable to create charges leading to 40 V and 100 μW peaks from single water drops landing on the upper artificial leaf surface whereas the lower silicone surface generated charges from the impact with the plant leaf.^52^ This does not only allow to use two energy sources but the possibility to harvest energy from rain drops compensates the typical decrease of energy output of solid–solid triboelectrification in high humidity leading to charge dissipation. Although this is reversible when the surfaces dry,^48,49^ the additional droplet energy harvesting in one device helps to increase and maintain the power output with little additional materials.

We also observed that a third energy source can be harvested by the plant tissue without significant modification, that is radio frequency (RF) radiation when the plant is connected as a receiving Marconi antenna to the energy harvesting circuits and a ground connection provides the potential difference.^51^ Here, the fact that the plant tissue is an ionic conductor acting as a “water or ionic antenna” is used and the ion current oscillates in the tissue when exposed to RF radiation.^123–125^ Combining these intrinsic properties with further tissue modification by 100 μm thin silicone coatings on the leaf surface, allowed to connect two ivy or Ficus plants in a way that uncoated leaves touch silicone-coated leaves when moving in the wind and this allows harvesting wind energy without installing artificial leaves.^51^ The RF and wind energy harvesting mode can be used simultaneously increasing the overall power output and enabling to power a wireless sensor node from RF and wind.

One of the key factors affecting the energy harvesting are the materials as they determine functionality from charge formation, to the aeroelastic properties and the effective contact area for charge formation. Moreover, the biodegradability and interactions of the artificial components with the plant ecosystem is an important aspect to address in the future. Achieving biodegradable artificial leaves for energy harvesting by triboelectric effects depends on suitable biodegradable materials that can generate sufficient charges, in quantities comparable to those generated on silicones or fluorinated polymers. The combination of efficient charge generation and on-demand environmental biodegradability remains a challenge that requires further research. Thus, tuning materials through their surface and bulk chemistry will be the key tool to expand energy harvesting capability of the systems and their application as autonomous micro power sources. Fig. 2(h) summarizes the design loop that the artificial leaves may go through for further improving their capabilities combining synthesis, and testing, as well as predictions through modelling *e.g.*, the mechanical properties of the composite materials. Further testing requires also evaluating the effects of the environment like changing weather conditions, frost, heat, dust, and changes in materials and plants over time under outdoor conditions are general aspects that need further understanding.

## 4. Advantages and challenges of a biohybrid approach

### 4.1. Advantages of a biohybrid approach

As demonstrated in our examples above, the perceived limitations of using plant-derived materials or even living plants in robotics, energy harvesting, and sensing can instead lead to significant advantages.

A clear advantage of learning to utilize materials intrinsically present in the environment for technical purposes such as energy harvesting or actuation is the reduction of the need to introduce entirely artificial systems into an ecosystem. For example, using the plant as a half electrode of a TENG reduces the need for an artificial material by at least 50% (or more using the leaf coatings presented above). Another example is the reuse of actuation mechanisms developed by plant fruits and seeds in a small-scale robot like the HybriBot: this approach reduces the need to manufacture artificial actuators at that scale, which can be challenging. This is especially important when plant-like functions should be accomplished by artificial and robotic systems, like seed dispersal. To this purpose, we have recently shown the first example of hybrid sustainable self-dispersing machine inspired by wild oat fruits, which use dead plant tissues as biological actuator and made with 100% biodegradable materials.^44^ This approach can lead to a new concept of “biohybrid robot” and can potentially be exploited for many other plant-like machines inspired by seeds and fruits that can autonomously adapt to complex multi-environments.

Second crucial advantage is that those materials that are directly derived from the living plant, usually do not harm the environment and ecosystem, and it could reduce the potential pollution introduced by artificial systems. While not all applications can avoid artificial materials, the process of learning how to use living or non-living plant-derived tissue in artificial or biohybrid devices can lead to new applications. This is a beginning field of research and especially combining them with electronics, robotics, approaches from nanotechnology and synthetic biology could add functionality to devices but also to the organisms like the plant itself.^106,126–128^

We recently powered for example an ion delivery system,^53^ or ion pump, directly from the energy harvested from plant leaf motion in the wind. Such systems could also deliver larger molecules like hormones stimulating plant growth as shown previously by Bernacka-Woijcik *et al.*^129^ Moreover, it has been observed that insects and pests like *Halyomorpha halys* introduce specific vibration patterns and electrical charges on leaves that can be measured with electrodes in the tissue.^130^ Such system could lead to new pest sensing approaches but further research on the understanding of charge formation on leaves, the combination of electrodes and feasibility is required. Especially, the time-dependent interaction of the artificial system and the plant needs to be observed and how materials exposed and interacting with the tissue remain functional. A crucial point is thereby the biocompatibility and the sustainability of the artificial materials that are introduced that still should be as biodegradable and harmless as possible as mentioned above.

### 4.2. Challenges of a biohybrid approach

Despite the advantages of biohybrid systems, several challenges and limitations must be considered.

One major concern is that environmental factors such as humidity, temperature fluctuations, and biological degradation can affect the functionality of plant-hybrid machines over time due to the inherent variability of biological but also artificial materials exposed to the environment. This variability can lead to inconsistencies in performance, durability, and reliability compared to fully artificial systems. Moreover, while biohybrid approaches aim to reduce environmental impact, ensuring that artificial components introduced into ecosystems are biodegradable and sustainable is crucial, particularly for long-term applications.^126,128^ Integrating plant-based materials with advanced technologies, presents additional technical challenges related to fabrication, scalability, and control.^131^

For example, although HybriBot presented significant advantages, it relies on dead plant tissues as biological actuators and a key challenge of this approach is that the availability and consistency of these natural materials can vary significantly.^44^ Unlike synthetic actuators, which can be manufactured in a controlled and repeatable manner, sourcing and processing plant-derived actuators at scale presents logistical and standardization challenges.

Another example includes wind and leaf-powered ion pumps, but optimizing their efficiency and stability for real-world applications and varying environmental conditions like wind speed remains yet a challenge in the early stage of such technologies.^53^ Similarly, sensors embedded in plant tissues have been used to detect pest-induced vibrations and electrical charges, yet further studies are needed to understand charge formation on leaves and improve the integration of artificial electrodes with biological systems^130^ including outdoor performance. A major challenge is still that artificial materials and devices are often static and cannot dynamically respond and evolve functionality adapting to variable external conditions.

As this is still an emerging field, further interdisciplinary research is needed to optimize biohybrid designs, enhance functionality, and address these limitations.

## 5. Conclusions and outlook

This feature article highlights the main contributions of the authors in the field of plant-inspired and/or plant-hybrid machines, showing significant progress in soft robotics, energy harvesting, and sensing technologies.

We show how researchers are extracting key principles from nature, especially plants, to design materials and robots able to not only interact but also preserve the ecosystems.

We have focused on the “chemistry” behind plant-inspired and/or hybrid machines for the phytosphere, by highlighting the manufacturing processes and materials, and exploring the energy harvesting advancements. Moreover, we have highlighted the most recent advances for adapting a biohybrid approach in robotics and material science as well as advantages and current challenges of the approach.

Despite in the recent years a growing number of studies has been performed in the plant-inspired and hybrid robotics field, many aspects remain unexplored. For example, the authors have shown novel plant-inspired machines able to attach to plant leaves for targeted drug-delivery into plant vascular tissues.^33^ Although precision medicine is a well-known approach in humans (*i.e.*, *via* microneedle patches,^132,133^ “precision plant medicine” could create a novel market to precisely deliver pesticides, nutrients and/or fertilizers into plants, helping preserving the ecosystem and the phytosphere). A few studies have been performed in this direction,^132^ and it could be interesting in future studies combine plant-inspired micropatterned materials into soft robotic arms^134^ for precision agriculture applications. In addition, biohybrid plant-like machines are also a growing research field in which many works still have to come. It could be interesting in future studies combining different biohybrid approaches by extracting living plant tissues (*i.e.*, living chloroplasts^54^) and combining it with biodegradable and responsive materials. We believe that energy autonomy is a key aspect that should be integrated from the initial design stages of any technology. The more such technologies are applied in environmental contexts, the greater the need for methods that avoid introducing pollution, may biodegrade, and still generate electricity for compatibility with our electronic technologies.

The energy conversion technologies presented here provide an overview of research and application possibilities, drawing inspiration from plants and utilizing biohybrid components to achieve functionalities that would be difficult or impossible with purely artificial systems. Further development of new functional materials, processable at multiple scales (from micro to macro) with a broader catalogue of biodegradable, sustainable materials will drive further process in the field. Especially the opportunity to combine multimodal functionalities such as energy conversion, actuation, tailored degradation, without the need for complex control systems but realized through physical intelligence, that is achieved through materials chemistry and its dynamic adaptation to the environment will allow next upgrades of the presented systems. A crucial aspect is also testing the systems in real environment where complex interaction could affect functionality drastically. Therefore, it is required that lab-scale fabrication methods and material systems allow to realistic outdoor tests with enough relevant replicates. Considering these aspects, and near future developments, sustainable, energy-autonomous robotics will significantly contribute to a broad technological platform for environmental monitoring, approaches for sustainable agriculture and for facilitating ecological restoration through advanced biohybrid robotic interventions.

## Author contributions

All authors contributed equally to writing of this feature article.

## Data availability

No primary research results, software or code have been included and no new data were generated or analysed as part of this review.

## Conflicts of interest

There are no conflicts to declare.

## References

[cit1] Wang Z. L. (2013). ACS Nano.

[cit2] Vincent J. F. V., Bogatyreva O. A., Bogatyrev N. R., Bowyer A., Pahl A.-K. (2006). J. R. Soc., Interface.

[cit3] He Q., Tang T., Zeng Y., Iradukunda N., Bethers B., Li X., Yang Y. (2024). Adv. Funct. Mater..

[cit4] Chen G., Liang X., Zhang P., Lin S., Cai C., Yu Z., Liu J. (2022). Adv. Funct. Mater..

[cit5] Gilbert C., Tang T.-C., Ott W., Dorr B. A., Shaw W. M., Sun G. L., Lu T. K., Ellis T. (2021). Nat. Mater..

[cit6] Mazzolai B., Beccai L., Mattoli V. (2014). Front. Bioeng. Biotechnol..

[cit7] Speck T., Cheng T., Klimm F., Menges A., Poppinga S., Speck O., Tahouni Y., Tauber F., Thielen M. (2023). MRS Bull..

[cit8] Fiorello I., Del Dottore E., Tramacere F., Mazzolai B. (2020). Bioinspiration Biomimetics.

[cit9] Burris J. N., Lenaghan S. C., Stewart C. N. (2018). Plant Cell Rep..

[cit10] Speck T. , Bold G., Masselter T., Poppinga S., Schmier S., Thielen M. and Speck O., in Plant Biomechanics: From Structure to Function at Multiple Scales, ed. A. Geitmann and J. Gril, Springer International Publishing, Cham, 2018, pp. 399–433

[cit11] Mazzolai B., Mariani S., Ronzan M., Cecchini L., Fiorello I., Cikalleshi K., Margheri L. (2021). Front. Robot. AI.

[cit12] Mazzolai B., Tramacere F., Fiorello I., Margheri L. (2020). Front. Robot. AI.

[cit13] Ledger S. E. H., Loh J., Almond R., Böhm M., Clements C. F., Currie J., Deinet S., Galewski T., Grooten M., Jenkins M., Marconi V., Painter B., Scott-Gatty K., Young L., Hoffmann M., Freeman R., McRae L. (2023). npj Biodivers..

[cit14] Yang T., Chen Y., Wang X.-X., Dai C.-C. (2013). Symbiosis.

[cit15] Hartmann F., Baumgartner M., Kaltenbrunner M. (2021). Adv. Mater..

[cit16] Chellapurath M., Khandelwal P. C., Schulz A. K. (2023). Front. Robot. AI.

[cit17] Mazzolai B., Laschi C. (2020). Sci. Robot..

[cit18] Kim S., Laschi C., Trimmer B. (2013). Trends Biotechnol..

[cit19] Ćurković P., Milvić D. (2024). Interdiscip. Descr. Complex Syst..

[cit20] Trimmer B. A. , Takesian A. E., Sweet B. M., Rogers C. B., Hake D. C. and Rogers D. J., in 7th international symposium on technology and the mine problem, Monterey, CA: Mine Warfare Association, 2006, vol. 1, pp. 1–10

[cit21] Xie Z., Yuan F., Liu J., Tian L., Chen B., Fu Z., Mao S., Jin T., Wang Y., He X., Wang G., Mo Y., Ding X., Zhang Y., Laschi C., Wen L. (2023). Sci. Robot..

[cit22] Li G., Wong T.-W., Shih B., Guo C., Wang L., Liu J., Wang T., Liu X., Yan J., Wu B. (2023). Nat. Commun..

[cit23] Picardi G., Chellapurath M., Iacoponi S., Stefanni S., Laschi C., Calisti M. (2020). Sci. Robot..

[cit24] Das R., Babu S. P. M., Visentin F., Palagi S., Mazzolai B. (2023). Sci. Rep..

[cit25] Ozkan-Aydin Y., Goldman D. I., Bhamla M. S. (2021). Proc. Natl. Acad. Sci. U. S. A..

[cit26] Rafsanjani A., Zhang Y., Liu B., Rubinstein S. M., Bertoldi K. (2018). Sci. Robot..

[cit27] Ruotolo W., Brouwer D., Cutkosky M. R. (2021). Sci. Robot..

[cit28] Romano D., Wahi A., Miraglia M., Stefanini C. (2022). Machines.

[cit29] Li L., Nagy M., Graving J. M., Bak-Coleman J., Xie G., Couzin I. D. (2020). Nat. Commun..

[cit30] Salazar J. , Cai L., Cook B. and Rus D., in 2022 IEEE/OES Autonomous Underwater Vehicles Symposium (AUV), IEEE, 2022, pp. 1–6

[cit31] De Croon G. C. H. E., Dupeyroux J. J. G., Fuller S. B., Marshall J. A. R. (2022). Sci. Robot..

[cit32] Ma K. Y., Chirarattananon P., Fuller S. B., Wood R. J. (2013). Science.

[cit33] Fiorello I., Meder F., Mondini A., Sinibaldi E., Filippeschi C., Tricinci O., Mazzolai B. (2021). Commun. Mater..

[cit34] Fiorello I. , Meder F., Tricinci O., Filippeschi C. and Mazzolai B., in Biomimetic and Biohybrid Systems, ed. U. Martinez-Hernandez, V. Vouloutsi, A. Mura, M. Mangan, M. Asada, T. J. Prescott and P. F. M. J. Verschure, Springer International Publishing, Cham, 2019, vol. 11556, pp. 122–133

[cit35] Fiorello I., Tricinci O., Naselli G. A., Mondini A., Filippeschi C., Tramacere F., Mishra A. K., Mazzolai B. (2020). Adv. Funct. Mater..

[cit36] Naselli G. A., Scharff R. B. N., Thielen M., Visentin F., Speck T., Mazzolai B. (2024). Adv. Intell. Syst..

[cit37] Tauber F. J., Auth P., Teichmann J., Scherag F. D., Speck T. (2022). Biomimetics.

[cit38] Cecchini L., Mariani S., Ronzan M., Mondini A., Pugno N. M., Mazzolai B. (2023). Adv. Sci..

[cit39] Luo D., Maheshwari A., Danielescu A., Li J., Yang Y., Tao Y., Sun L., Patel D. K., Wang G., Yang S. (2023). Nature.

[cit40] Fiorello I. , Margheri L., Filippeschi C. and Mazzolai B., in 2022 IEEE 5th International Conference on Soft Robotics (RoboSoft), IEEE, 2022, pp. 255–260

[cit41] Mariani S., Cikalleshi K., Ronzan M., Filippeschi C., Naselli G. A., Mazzolai B. (2024). Small.

[cit42] Cikalleshi K., Nexha A., Kister T., Ronzan M., Mondini A., Mariani S., Kraus T., Mazzolai B. (2023). Sci. Adv..

[cit43] Yang J., Zhang H., Berdin A., Hu W., Zeng H. (2023). Adv. Sci..

[cit44] Fiorello I., Ronzan M., Speck T., Sinibaldi E., Mazzolai B. (2024). Adv. Mater..

[cit45] Kim B. H., Li K., Kim J.-T., Park Y., Jang H., Wang X., Xie Z., Won S. M., Yoon H.-J., Lee G., Jang W. J., Lee K. H., Chung T. S., Jung Y. H., Heo S. Y., Lee Y., Kim J., Cai T., Kim Y., Prasopsukh P., Yu Y., Yu X., Avila R., Luan H., Song H., Zhu F., Zhao Y., Chen L., Han S. H., Kim J., Oh S. J., Lee H., Lee C. H., Huang Y., Chamorro L. P., Zhang Y., Rogers J. A. (2021). Nature.

[cit46] Ching T., Lee J. Z. W., Win S. K. H., Win L. S. T., Sufiyan D., Lim C. P. X., Nagaraju N., Toh Y.-C., Foong S., Hashimoto M. (2024). Sci. Robot..

[cit47] Fiorello I. , Mondini A. and Mazzolai B., in 2023 IEEE International Conference on Soft Robotics (RoboSoft), IEEE, 2023, pp. 1–7

[cit48] Meder F., Thielen M., Mondini A., Speck T., Mazzolai B. (2020). Energy Technol..

[cit49] Meder F., Armiento S., Naselli G. A., Mondini A., Speck T., Mazzolai B. (2024). Bioinspiration Biomimetics.

[cit50] Meder F., Armiento S., Naselli G. A., Thielen M., Speck T., Mazzolai B. (2021). Bioinspiration Biomimetics.

[cit51] Meder F., Mondini A., Visentin F., Zini G., Crepaldi M., Mazzolai B. (2022). Energy Environ. Sci..

[cit52] Armiento S., Meder F., Mazzolai B. (2023). IEEE Robot. Autom. Lett..

[cit53] Armiento S., Bernacka-Wojcik I., Dar A. M., Meder F., Stavrinidou E., Mazzolai B. (2024). Bioinspiration Biomimetics.

[cit54] Yu K., Feng Z., Du H., Xin A., Lee K. H., Li K., Su Y., Wang Q., Fang N. X., Daraio C. (2021). Proc. Natl. Acad. Sci. U. S. A..

[cit55] Del Dottore E., Mondini A., Rowe N., Mazzolai B. (2024). Sci. Robot..

[cit56] Chen W. (2022). Chem. Eng. J..

[cit57] De Pascali C., Naselli G. A., Palagi S., Scharff R. B. N., Mazzolai B. (2022). Sci. Robot..

[cit58] Lee K., Yang D., Park S. H., Kim R. H. (2006). Polym. Adv. Technol..

[cit59] Lee Y., Kim J., Bozuyuk U., Dogan N. O., Khan M. T. A., Shiva A., Wild A., Sitti M. (2023). Adv. Mater..

[cit60] Yang W., Wang Z., Wang X., Yu T., Xie S., Ge Z. (2023). Opt. Laser Technol..

[cit61] Bastola A. K., Rodriguez N., Behl M., Soffiatti P., Rowe N. P., Lendlein A. (2021). Mater. Des..

[cit62] Viola F. A., Maksimovic K., Cataldi P., Rinaldi C., Stucchi E., Melloni F., Athanassiou A., Caironi M. (2024). Mater. Today Bio.

[cit63] Rumley E. H., Preninger D., Shagan Shomron A., Rothemund P., Hartmann F., Baumgartner M., Kellaris N., Stojanovic A., Yoder Z., Karrer B., Keplinger C., Kaltenbrunner M. (2023). Sci. Adv..

[cit64] Armada-Moreira A., Dar A. M., Zhao Z., Cea C., Gelinas J., Berggren M., Costa A., Khodagholy D., Stavrinidou E. (2023). Sci. Adv..

[cit65] Bae J.-Y., Hwang G.-S., Kim Y.-S., Jeon J., Chae M., Kim J.-W., Lee S., Kim S., Lee S.-H., Choi S.-G., Lee J.-Y., Lee J.-H., Kim K.-S., Park J.-H., Lee W.-J., Kim Y.-C., Lee K.-S., Kim J., Lee H., Hyun J. K., Kim J.-Y., Kang S.-K. (2024). Nat. Electron..

[cit66] van Laake L. C., Overvelde J. T. B. (2024). Commun. Mater..

[cit67] Greenman J., Holland O., Kelly I., Kendall K., McFarland D., Melhuish C. (2003). Mechatronics.

[cit68] Nie Z.-Z., Wang M., Yang H. (2024). Commun. Chem..

[cit69] Miskin M. Z., Cortese A. J., Dorsey K., Esposito E. P., Reynolds M. F., Liu Q., Cao M., Muller D. A., McEuen P. L., Cohen I. (2020). Nature.

[cit70] Chen X., Jang B., Ahmed D., Hu C., De Marco C., Hoop M., Mushtaq F., Nelson B. J., Pané S. (2018). Adv. Mater..

[cit71] Hines L., Petersen K., Lum G. Z., Sitti M. (2017). Adv. Mater..

[cit72] Aubin C. A., Heisser R. H., Peretz O., Timko J., Lo J., Helbling E. F., Sobhani S., Gat A. D., Shepherd R. F. (2023). Science.

[cit73] Shepherd R. F., Stokes A. A., Freake J., Barber J., Snyder P. W., Mazzeo A. D., Cademartiri L., Morin S. A., Whitesides G. M. (2013). Angew. Chem., Int. Ed..

[cit74] Aubin C. A., Gorissen B., Milana E., Buskohl P. R., Lazarus N., Slipher G. A., Keplinger C., Bongard J., Iida F., Lewis J. A. (2022). Nature.

[cit75] Xiong J., Li X., He Z., Shi Y., Pan T., Zhu G., Lu D., Xin H. (2024). Light: Sci. Appl..

[cit76] Gu M., Echtermeyer T. J. (2024). Small.

[cit77] Qin J., Chu K., Huang Y., Zhu X., Hofkens J., He G., Parkin I. P., Lai F., Liu T. (2021). Energy Environ. Sci..

[cit78] Li C., He Q., Wang Y., Wang Z., Wang Z., Annapooranan R., Latz M. I., Cai S. (2022). Nat. Commun..

[cit79] Shin B., Ha J., Lee M., Park K., Park G. H., Choi T. H., Cho K.-J., Kim H.-Y. (2018). Sci. Robot..

[cit80] Luo D., Maheshwari A., Danielescu A., Li J., Yang Y., Tao Y., Sun L., Patel D. K., Wang G., Yang S., Zhang T., Yao L. (2023). Nature.

[cit81] Cecchini L., Mariani S., Ronzan M., Mondini A., Pugno N. M., Mazzolai B. (2023). Adv. Sci..

[cit82] Yang X., Lan L., Pan X., Di Q., Liu X., Li L., Naumov P., Zhang H. (2023). Nat. Commun..

[cit83] Kanik M., Orguc S., Varnavides G., Kim J., Benavides T., Gonzalez D., Akintilo T., Tasan C. C., Chandrakasan A. P., Fink Y., Anikeeva P. (2019). Science.

[cit84] Fang S., Hu Y. H. (2024). Chem. Commun..

[cit85] Meder F., Must I., Sadeghi A., Mondini A., Filippeschi C., Beccai L., Mattoli V., Pingue P., Mazzolai B. (2018). Adv. Funct. Mater..

[cit86] Armiento S., Filippeschi C., Meder F., Mazzolai B. (2022). Commun. Mater..

[cit87] Stark R. E. and Tian S., Annual Plant Reviews: Biology of the Plant Cuticle, John Wiley & Sons, Ltd, 2006, vol. 23, pp. 126–144

[cit88] Jetter R. , Kunst L. and Samuels A. L., Annual Plant Reviews: Biology of the Plant Cuticle, John Wiley & Sons, Ltd, 2006, vol. 23, pp. 145–181

[cit89] Li S., Zhang J., He J., Liu W., Wang Y., Huang Z., Pang H., Chen Y. (2023). Adv. Sci..

[cit90] Barthlott W., Neinhuis C. (1997). Planta.

[cit91] Barthlott W., Mail M., Bhushan B., Koch K. (2017). Nano-Micro Lett..

[cit92] Zou H., Guo L., Xue H., Zhang Y., Shen X., Liu X., Wang P., He X., Dai G., Jiang P., Zheng H., Zhang B., Xu C., Wang Z. L. (2020). Nat. Commun..

[cit93] Seol M.-L., Woo J.-H., Lee D.-I., Im H., Hur J., Choi Y.-K. (2014). Small.

[cit94] Jin S., Wang Y., Motlag M., Gao S., Xu J., Nian Q., Wu W., Cheng G. J. (2018). Adv. Mater..

[cit95] Liu S., Liu X., Zhou G., Qin F., Jing M., Li L., Song W., Sun Z. (2020). Nat. Commun..

[cit96] Zheng Y., Liu T., Wu J., Xu T., Wang X., Han X., Cui H., Xu X., Pan C., Li X. (2022). Adv. Mater..

[cit97] Shi X., Wei Y., Yan R., Hu L., Zhi J., Tang B., Li Y., Yao Z., Shi C., Yu H.-D., Huang W. (2023). Nano Energy.

[cit98] Begum S. R., Chandrasekhar A. (2024). iScience.

[cit99] Wang L., Xu H., Huang F., Tao X., Ouyang Y., Zhou Y., Mo X. (2022). Nanomaterials.

[cit100] Lan L., Xiong J., Gao D., Li Y., Chen J., Lv J., Ping J., Ying Y., Lee P. S. (2021). ACS Nano.

[cit101] Jie Y., Jia X., Zou J., Chen Y., Wang N., Wang Z. L., Cao X. (2018). Adv. Energy Mater..

[cit102] Choi D., Kim D. W., Yoo D., Cha K. J., La M., Kim D. S. (2017). Nano Energy.

[cit103] Li X., Jiang C., Yao Y., Zhang Q., Dai S., Ying Y., Ping J. (2021). Small.

[cit104] Wu H., Chen Z., Xu G., Xu J., Wang Z., Zi Y. (2020). ACS Appl. Mater. Interfaces.

[cit105] Kim D. W., Kim S.-W., Jeong U. (2018). Adv. Mater..

[cit106] Dufil G., Bernacka-Wojcik I., Armada-Moreira A., Stavrinidou E. (2022). Chem. Rev..

[cit107] Parker D., Dar A. M., Armada-Moreira A., Bernacka Wojcik I., Rai R., Mantione D., Stavrinidou E. (2024). ACS Appl. Mater. Interfaces.

[cit108] Fan F.-R., Tian Z.-Q., Wang Z. L. (2012). Nano Energy.

[cit109] Yang Y., Zhu G., Zhang H., Chen J., Zhong X., Lin Z.-H., Su Y., Bai P., Wen X., Wang Z. L. (2013). ACS Nano.

[cit110] Chen B., Yang Y., Wang Z. L. (2018). Adv. Energy Mater..

[cit111] Zheng Q., Zou Y., Zhang Y., Liu Z., Shi B., Wang X., Jin Y., Ouyang H., Li Z., Wang Z. L. (2016). Sci. Adv..

[cit112] Yao C., Yin X., Yu Y., Cai Z., Wang X. (2017). Adv. Funct. Mater..

[cit113] Wang R., Gao S., Yang Z., Li Y., Chen W., Wu B., Wu W. (2018). Adv. Mater..

[cit114] Jiang W., Li H., Liu Z., Li Z., Tian J., Shi B., Zou Y., Ouyang H., Zhao C., Zhao L., Sun R., Zheng H., Fan Y., Wang Z. L., Li Z. (2018). Adv. Mater..

[cit115] Zhang R., Olin H. (2020). EcoMat.

[cit116] Li X., Yang Q., Ren D., Li Q., Yang H., Zhang X., Xi Y. (2024). Nanoscale Adv..

[cit117] Meder F., Naselli G. A., Mazzolai B. (2022). Front. Plant Sci..

[cit118] Zhu W., Hu C., Bowen C. R., Wang Z. L., Yang Y. (2022). Nano Energy.

[cit119] McCarthy J. M., Watkins S., Deivasigamani A., John S. J. (2016). J. Sound Vib..

[cit120] Dong J., Ru Fan F., Tian Z.-Q. (2021). Nanoscale.

[cit121] Hasan M. A. M., Zhang T., Wu H., Yang Y. (2022). Adv. Energy Mater..

[cit122] Lin S., Chen X., Wang Z. L. (2022). Chem. Rev..

[cit123] Kaur S., Vian A., Chandel S., Singh H. P., Batish D. R., Kohli R. K. (2021). Rev. Environ. Sci. Biotechnol..

[cit124] Huang Y., Xing L., Song C., Wang S., Elhouni F. (2021). IEEE Open J. Antennas Propag..

[cit125] Xing L. , Huang Y., Alja’afreh S. S. and Boyes S. J., in 2012 Loughborough Antennas & Propagation Conference (LAPC), 2012, pp. 1–4

[cit126] Meder F., Baytekin B., Dottore E. D., Meroz Y., Tauber F., Walker I., Mazzolai B. (2022). Bioinspiration Biomimetics.

[cit127] Wang Q., Hu Z., Li Z., Liu T., Bian G. (2023). Adv. Mater..

[cit128] Giraldo J. P., Wu H., Newkirk G. M., Kruss S. (2019). Nat. Nanotechnol..

[cit129] Bernacka-Wojcik I., Huerta M., Tybrandt K., Karady M., Mulla M. Y., Poxson D. J., Gabrielsson E. O., Ljung K., Simon D. T., Berggren M., Stavrinidou E. (2019). Small.

[cit130] Armiento S. , Meder F. and Mazzolai B., in Biomimetic and Biohybrid Systems, ed. F. Meder, A. Hunt, L. Margheri, A. Mura and B. Mazzolai, Springer, Nature Switzerland, Cham, 2023, pp. 303–317

[cit131] Nguyen P. Q., Courchesne N.-M. D., Duraj-Thatte A., Praveschotinunt P., Joshi N. S. (2018). Adv. Mater..

[cit132] Prausnitz M. R. (2017). Annu. Rev. Chem. Biomol. Eng..

[cit133] Primavera R., Kevadiya B. D., Swaminathan G., Wilson R. J., De Pascale A., Decuzzi P., Thakor A. S. (2020). Nanomaterials.

[cit134] Elfferich J. F., Dodou D., Santina C. D. (2022). IEEE Access.

